# An interpretable stacked ensemble framework for evaluating respiratory rehabilitation outcomes under traditional Chinese medicine-integrated care: a multicenter retrospective cohort study

**DOI:** 10.3389/fmed.2026.1808176

**Published:** 2026-05-28

**Authors:** Yinglian Li, Zhiping Wu

**Affiliations:** Jinjiang Municipal Hospital (Shanghai sixth People’s Hospital Fujian), Jinjiang, Fujian, China

**Keywords:** interpretable machine learning, respiratory rehabilitation, stacked ensemble learning, traditional Chinese medicine, treatment outcome

## Abstract

**Background:**

Evaluating treatment effectiveness in patients undergoing respiratory rehabilitation integrated with Traditional Chinese Medicine (TCM) remains challenging because recovery trajectories are clinically heterogeneous, interventions are multimodal, and treatment effects may be nonlinear. Conventional assessment strategies often rely on score changes or threshold-based criteria and therefore provide limited support for individualized evaluation and clinically grounded interpretation.

**Methods:**

This multicenter retrospective cohort study included 1,483 patients who received respiratory rehabilitation combined with TCM-based interventions. Predictor variables covered demographic and clinical characteristics, disease severity, rehabilitation prescription and implementation intensity, TCM intervention features, laboratory and physiological indicators, and baseline functional status. Data were randomly divided into training and test sets at a ratio of 7:3 for internal validation. Within the training set, feature selection was performed using the least absolute shrinkage and selection operator (LASSO), and the selected variables were entered into a stacked ensemble framework. Model performance was evaluated using discrimination and calibration metrics. Model interpretability was assessed using SHapley Additive exPlanations (SHAP) to quantify both global and individual-level feature contributions.

**Results:**

The stacked ensemble model achieved the best predictive performance in the independent test set, with an area under the receiver operating characteristic curve of 0.903, outperforming all single learners. SHAP analysis indicated that baseline functional capacity and symptom burden were dominant predictors of rehabilitation outcomes. Measures reflecting TCM intervention intensity, particularly exercise frequency and duration of herbal exposure, also contributed substantially to prediction and showed nonlinear dose–response patterns. These findings suggest that TCM-related treatment characteristics provided additional clinically interpretable information beyond baseline patient status alone.

**Conclusion:**

The proposed interpretable stacked ensemble framework provides a transparent approach for evaluating respiratory rehabilitation outcomes under TCM-integrated care. By identifying nonlinear effects and patient-specific drivers of treatment response, the model may support more individualized outcome assessment and more informed clinical decision-making. Further validation across independent external cohorts is warranted before broader clinical implementation.

## 1 Introduction

Respiratory diseases, including chronic obstructive pulmonary disease (COPD), asthma, and persistent respiratory symptoms after infection, are often characterized by prolonged clinical courses, substantial disease burden, and marked inter-individual variability in both presentation and recovery (1, 2). Accordingly, the evaluation of rehabilitation outcomes has gradually moved beyond reliance on a single pulmonary function indicator toward a multidimensional framework that also considers exercise tolerance, symptom burden, quality of life, and functional recovery (3). Within this broader rehabilitation context, non-pharmacological strategies, including Traditional Chinese Medicine (TCM) therapies and traditional exercise-based interventions, have been increasingly incorporated into respiratory care. Recent systematic reviews and evidence syntheses suggest that selected TCM-based exercise approaches may improve pulmonary function, exercise capacity, and symptom control in patients with COPD, although the strength of evidence still varies across intervention types and study quality (4, 5). However, in real-world practice, substantial differences in baseline status, comorbid conditions, and overall health profiles contribute to marked heterogeneity in treatment response. As a result, determining which patients are most likely to benefit, how much improvement can reasonably be expected, and when rehabilitation outcomes should be assessed remains a persistent challenge in both rehabilitation medicine and TCM-integrated care.

At present, mainstream outcome assessment still depends largely on score changes or threshold-based judgments and is therefore vulnerable to variation related to observation timing, inter-rater inconsistency, patient effort, and the influence of concurrent diseases. Improvements in symptoms, physical endurance, psychological status, and functional performance may also emerge asynchronously within the same individual. Conventional assessment approaches therefore often show instability at the individual level because they do not adequately integrate multidimensional information or apply transparent weighting across outcome domains. This problem is particularly relevant in TCM-integrated rehabilitation, where treatment-related information such as syndrome differentiation, herbal prescriptions, dosage intensity, treatment duration, and adherence may contribute meaningfully to therapeutic effectiveness but is often documented in unstructured or semi-structured clinical narratives, making it difficult to standardize and incorporate into conventional statistical frameworks capable of generating reproducible evidence (6). Recent reviews of explainable artificial intelligence further indicate that, in healthcare settings, prediction systems lacking interpretability and traceable feature attribution are less likely to gain clinician trust or support real-world decision-making (7, 8).

In recent years, machine-learning approaches have been increasingly applied to respiratory outcome prediction and risk stratification. Nevertheless, many existing studies remain constrained by limited handling of complex feature interactions, insufficient robustness in high-dimensional settings, and an ongoing risk of overfitting when model development and validation are not adequately separated. In this context, ensemble learning has attracted growing attention because it can combine the complementary strengths of multiple algorithms and often achieve more stable predictive discrimination than individual learners alone (9). Recent studies in respiratory and pneumonia-related prediction have likewise shown that ensemble architectures can improve classification performance, while feature-attribution tools help identify the variables driving model output (10). When such models are coupled with SHapley Additive exPlanations (SHAP), the contribution of individual predictors can be quantified at both the global and patient-specific levels, thereby improving transparency and making model output more clinically interpretable (11).

Against this background, the present study aimed to develop an interpretable stacked learning framework for evaluating rehabilitation outcomes in patients with respiratory diseases receiving TCM-integrated interventions within a retrospective real-world cohort. The proposed framework incorporated demographic and clinical characteristics, disease severity, rehabilitation prescription and implementation intensity, TCM intervention features, laboratory and physiological indicators, and baseline functional status within a unified analytical structure. The dataset was divided into training and test subsets at a ratio of 7:3 for internal validation, and model performance in the independent test set was assessed using discrimination and calibration metrics, including the receiver operating characteristic curve and the area under the curve. SHAP was further used to generate both global and individual-level explanations, with the aim of identifying the predictors and predictor combinations most relevant to distinguishing improvement from non-improvement after rehabilitation. By combining predictive robustness with interpretability, this study sought to provide a more transparent and reproducible approach to the evaluation of TCM-integrated respiratory rehabilitation outcomes and to establish a basis for subsequent external validation and clinical verification.

## 2 Materials and methods

### 2.1 Study design and cohort

This multicenter retrospective cohort study was conducted to develop and internally validate an interpretable machine-learning framework for evaluating rehabilitation effectiveness after Traditional Chinese Medicine (TCM)-integrated interventions in patients with respiratory diseases. Clinical records were obtained from the Departments of Rehabilitation Medicine and Traditional Chinese Medicine of four tertiary hospitals in Fujian Province, China. Jinjiang Municipal Hospital (Shanghai Sixth People’s Hospital Fujian) served as the lead institution, and the remaining participating hospitals joined the study through formal institutional authorization for retrospective data collaboration and de-identified data sharing under a unified study framework. Both directly managed cases and referred cases were eligible for screening.

Patients were included if they met all of the following criteria: (1) a documented diagnosis of a respiratory disease relevant to rehabilitation management, including chronic obstructive pulmonary disease, asthma, post-infectious persistent respiratory symptoms, or other respiratory rehabilitation-related conditions; (2) receipt of at least one TCM-related intervention during the index rehabilitation course; and (3) availability of both baseline assessment data and end-of-course outcome evaluation. Patients were excluded if key baseline predictors or primary outcome data were missing, if the rehabilitation course did not meet the predefined minimum observation window, or if duplicate, contradictory, or otherwise non-resolvable records remained after data cleaning.

**Figure 1:**
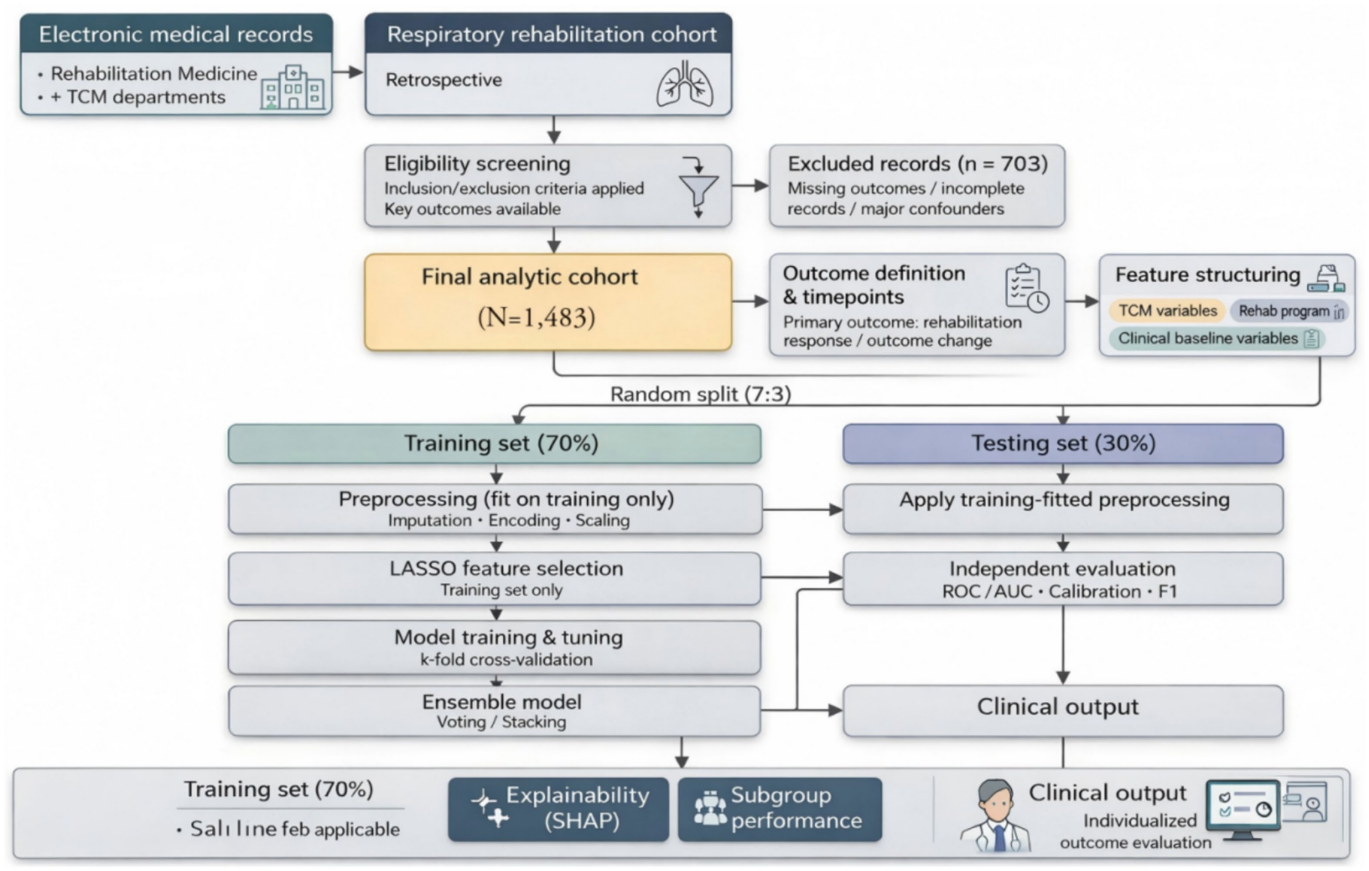
Flowchart of patient screening, cohort construction, and stratified internal train-test split.

A total of 2,186 cases were initially screened. After application of the uniform inclusion and exclusion criteria, 703 cases were excluded and 1,483 eligible cases were retained for model development and validation. The cohort construction process is presented in Figure 1.

The final analytic cohort was divided into training and test sets at a ratio of 7:3 using a stratified random split based on the primary outcome. This approach was selected to preserve outcome balance between subsets while maintaining a sufficiently large development sample for feature selection, model fitting, and hyperparameter optimization, together with an independent hold-out set for internal performance evaluation. A leave-one-subject-out cross-validation strategy was not adopted because the present study addressed cohort-level clinical outcome prediction rather than repeated within-subject classification. In addition, because the participating hospitals differed in sample size, case-mix structure, and treatment-pattern distribution, no single center could serve as a sufficiently stable and independent external validation cohort without substantially reducing the robustness of model development. Accordingly, the present study should be interpreted as an internally validated multicenter prediction study rather than a formal external validation study.

Within the modeling framework, the training set was used for data preprocessing, feature engineering, variable selection, model fitting, and hyperparameter tuning. The test set remained fully isolated from model development and was used exclusively for final evaluation of discrimination, calibration, and classification performance, thereby reducing the risk of information leakage and overly optimistic estimates. To account for possible between-center heterogeneity within the internal validation framework, hospital identifier was retained as a structured variable in the analytic dataset.

For cohort construction, a uniform index date was defined as the date on which the patient entered the rehabilitation assessment pathway or completed the baseline evaluation. Baseline predictors were collected at or before the index date, whereas intervention-related and implementation-intensity variables were captured during the rehabilitation course. Outcome assessment was performed at the end of the predefined observation window using harmonized criteria across centers to improve temporal comparability.

This study was conducted under the coordination of Jinjiang Municipal Hospital (Shanghai Sixth People’s Hospital Fujian), whose Ethics Committee served as the primary reviewing body and approved the study protocol (Approval No. jjsyyll-2026-043). The remaining participating hospitals provided institutional authorization for retrospective data collaboration and de-identified data sharing within the scope of the unified study protocol. All records were anonymized before extraction and analysis. Because the study used existing de-identified clinical data and involved no direct patient contact or intervention, the requirement for written informed consent was waived or handled in accordance with local institutional regulations at the participating centers.

### 2.2 TCM interventions, rehabilitation documentation, and candidate predictor framework

To ensure comparability and reproducibility in this multicenter retrospective study, TCM interventions and rehabilitation programs were extracted using predefined coding rules and structured documentation procedures. These variables constituted the core exposure and treatment-intensity components of the candidate predictor framework. TCM-related interventions included both pharmacological and non-pharmacological modalities. Pharmacological treatments comprised herbal decoctions or prescriptions and Chinese patent medicines. Non-pharmacological interventions included acupuncture, moxibustion, Tuina, and traditional exercise therapy.

Because real-world rehabilitation practice involves substantial variation in prescription composition, treatment duration, and adherence, the study captured not only whether a given intervention was administered but also the extent of its implementation. Accordingly, both categorical and continuous indicators were extracted to reflect potential dose–response patterns. These variables included, where applicable, days of herbal use, dose-intensity level, number of acupuncture or moxibustion sessions, and weekly frequency of TCM exercise therapy.

To preserve the clinical logic of syndrome differentiation in TCM while improving model usability, baseline syndrome information was standardized during data extraction. Syndrome categories were assigned on the basis of explicitly documented physician diagnoses in the medical records. For modeling purposes, syndrome-related information was represented in two complementary forms: a primary syndrome-category variable and a group of binary syndrome-element indicators. In addition, individual symptom items were aggregated to generate a syndrome severity score reflecting the baseline burden of syndrome manifestation.

Adherence variables were derived preferentially from objective and verifiable sources. Medication adherence was evaluated using pharmacy dispensing or refill records in combination with nursing documentation. Adherence to rehabilitation training was determined from therapist attendance logs, rehabilitation implementation records, and exercise documentation. This strategy reduced reliance on subjective self-report and improved the consistency of retrospective measurement across centers.

Rehabilitation co-interventions were documented using three coordinated dimensions: prescription content, implementation intensity, and treatment duration. Prescription content covered the principal pulmonary rehabilitation components routinely used in clinical practice, including aerobic training, resistance training, breathing training, inspiratory muscle training, airway-clearance techniques, and self-management education. Implementation intensity was quantified using indicators such as weekly training time, training frequency, and the use of specialized rehabilitation devices. Treatment duration was defined as the number of days from baseline assessment to treatment completion, discharge, or attainment of predefined therapeutic goals.

To reduce inter-center variability during retrospective extraction, uniform variable definitions, coding rules, measurement units, and acquisition time points were applied across all participating sites. Hospital identifier and visit type were retained in the structured dataset to support assessment of center-related heterogeneity and clinical-context variation. For consistency between the narrative description and the data architecture, all candidate predictors were organized into the same six predefined domains used throughout the manuscript: demographic and clinical characteristics, disease severity, rehabilitation prescription and implementation intensity, TCM intervention features, laboratory and physiological indicators, and baseline functional status. Because the original intervention-coding table and the candidate-predictor table were partially overlapping, they were merged into a single revised predictor framework. The full predictor structure, together with variable definitions, coding rules, data sources, and acquisition time points, is presented in Table 1.

**Table 1:** Structured candidate predictor framework, including TCM intervention coding and rehabilitation co-intervention variables.

Domain	Predictor	Type	Unit/categories	Data source	Time Point
Demographic and clinical characteristics	Age	Continuous	Years	EMR registration	T0
Demographic and clinical characteristics	Sex	Binary	Male/female	EMR registration	T0
Demographic and clinical characteristics	BMI	Continuous	kg/m^2^	Nursing assessment	T0
Demographic and clinical characteristics	Smoking_status	Categorical	Never/former/current	Admission note	T0
Demographic and clinical characteristics	Visit_type	Categorical	Inpatient/outpatient	Visit record	T0
Demographic and clinical characteristics	Primary_diagnosis	Categorical	COPD / asthma / post-infectious / others	ICD + discharge diagnosis	T0
Demographic and clinical characteristics	CAT_baseline	Continuous	0–40	Scale record	T0
Demographic and clinical characteristics	mMRC_baseline	Ordinal	0–4	Scale record	T0
Demographic and clinical characteristics	Exacerbations_12m	Ordinal	0/1 / ≥2	EMR history	T0
Demographic and clinical characteristics	Comorbidity_Index (CCI)	Continuous	Score	EMR problem list	T0
Rehabilitation prescription and implementation intensity	Pulmonary_rehab	Binary	0/1	Rehabilitation prescription	During course
Rehabilitation prescription and implementation intensity	Rehab_course_length	Continuous	Days	Rehabilitation record	T1
Rehabilitation prescription and implementation intensity	Aerobic_training_min_perweek	Continuous	Min/week	Therapist log	During course
Rehabilitation prescription and implementation intensity	Resistance_training_sessions_perweek	Continuous	Sessions/week	Therapist log	During course
Rehabilitation prescription and implementation intensity	Breathing_training	Binary	0/1	Rehabilitation prescription/log	During course
Rehabilitation prescription and implementation intensity	IMT_use	Binary	0/1	Rehabilitation prescription/log	During course
Rehabilitation prescription and implementation intensity	Airway_clearance_technique	Binary	0/1	Rehabilitation prescription/log	During course
Rehabilitation prescription and implementation intensity	Education_selfmanagement	Binary	0/1	Nursing/therapist record	During course
TCM intervention features	Herbal_decoction	Binary	0/1	Medication orders + physician notes	During course
TCM intervention features	Chinese_patent_medicine	Binary	0/1	Medication orders	During course
TCM intervention features	Acupuncture	Binary	0/1	Procedure record	During course
TCM intervention features	Moxibustion	Binary	0/1	Procedure record	During course
TCM intervention features	Tuina	Binary	0/1	Procedure record	During course
TCM intervention features	TCM_exercise_therapy	Binary	0/1	Rehabilitation/TCM guidance note	During course
TCM intervention features	Herbal_days	Continuous	Days	Medication orders	During course
TCM intervention features	Herbal_dose_intensity	Ordinal	0–3	Medication orders	During course
TCM intervention features	Patent_med_days	Continuous	days	Medication orders	During course
TCM intervention features	Acupuncture_sessions	Continuous	Count	Procedure record	During course
TCM intervention features	Moxibustion_sessions	Continuous	Count	Procedure record	During course
TCM intervention features	TCM_exercise_sessions_perweek	Continuous	0–7	Therapist/nursing logs	During course
TCM intervention features	Syndrome_category	Categorical	Standardized categories	TCM physician note	T0
TCM intervention features	Syndrome_Qi_deficiency	Binary	0/1	TCM physician note	T0
TCM intervention features	Syndrome_Phlegm_dampness	Binary	0/1	TCM physician note	T0
TCM intervention features	Syndrome_Yin_deficiency	Binary	0/1	TCM physician note	T0
TCM intervention features	Syndrome_blood_stasis	Binary	0/1	TCM physician note	T0
TCM intervention features	Syndrome_heat_toxin	Binary	0/1	TCM physician note	T0
TCM intervention features	Syndrome_severity_score	Continuous	0–12	Structured extraction from notes	T0
TCM intervention features	Adherence_TCM_medication	Ordinal	0–3	Pharmacy + nursing notes	During course
TCM intervention features	Adherence_rehab_training	Ordinal	0–3	Therapist logs	During course
Laboratory and physiological indicators	Resting_SpO2	Continuous	%	Vital signs	T0
Laboratory and physiological indicators	Respiratory_rate	Continuous	Breaths/min	Vital signs	T0
Laboratory and physiological indicators	Heart_rate	Continuous	Bpm	Vital signs	T0
Laboratory and physiological indicators	NLR	Continuous	Ratio	Laboratory	T0
Baseline functional status	6MWD_baseline	Continuous	m	6-min walk test record	T0
Baseline functional status	Borg_post6MWT_baseline	Continuous	0–10	6-min walk test record	T0
Center	Hospital_ID	Categorical	1–4	EMR site field	T0

TCM, traditional Chinese medicine; EMR, electronic medical record; BMI, body mass index; COPD, chronic obstructive pulmonary disease; ICD, international classification of diseases; CAT, COPD assessment test; mMRC, modified Medical Research Council dyspnea scale; CCI, Charlson Comorbidity Index; IMT, inspiratory muscle training; SpO2, peripheral oxygen saturation; NLR, neutrophil-to-lymphocyte ratio; 6MWD, 6-min walk distance; T0, baseline; T1, end of rehabilitation course or end of predefined observation window.

### 2.3 Outcome definition and assessment schedule

The primary analytical endpoint was end-of-course rehabilitation response. Outcome assessment followed a predefined schedule. Baseline assessment (T0) was defined as the time of entry into the rehabilitation pathway or completion of the initial standardized evaluation. The primary post-treatment assessment (T1) was performed at the end of the rehabilitation course and was uniformly defined as 4 weeks ± 7 days. When available, an additional follow-up assessment at approximately 3 months after baseline (T2, ± 14 days) was collected for descriptive follow-up analysis; however, T2 data were not used to define the primary endpoint or to train the prediction models.

The primary outcome was dichotomized as Responder versus Non-responder according to whether clinically meaningful improvement was achieved between T0 and T1. A patient was classified as a Responder if at least one prespecified minimal clinically important difference (MCID) threshold was reached, defined as an increase of at least 30 m in 6-min walk distance (6MWD) or a decrease of at least 2 points in the COPD Assessment Test (CAT) score. This composite definition was selected because it captures two clinically relevant dimensions of respiratory rehabilitation effectiveness, namely functional exercise capacity and symptom burden, and is therefore suitable for integrated evaluation in routine rehabilitation settings. Patients who did not meet either threshold were classified as Non-responders.

Secondary outcomes were analyzed to further characterize treatment-related changes and included continuous or ordinal changes in 6MWD, CAT score, modified Medical Research Council (mMRC) dyspnea grade, resting peripheral oxygen saturation (SpO2), and Borg dyspnea score after the 6-min walk test. All outcomes were defined *a priori* using harmonized assessment criteria across centers. Detailed outcome definitions, measurement instruments, assessment time points, and responder thresholds are summarized in Table 2.

**Table 2:** Definitions of rehabilitation outcomes, assessment schedule, and responder thresholds.

Outcome level	Outcome name	Instrument/source	Assessment time points	Metric	Responder criteria /MCID threshold	Direction
Primary	Rehabilitation response (Responder)	Derived from functional and symptom change	T0 baseline; T1 end of course (4 weeks ± 7 days)	Binary (0/1)	Responder = Δ6MWD ≥ 30 m or ΔCAT ≤ − 2 points from T0 to T1	Higher 6MWD; lower CAT
Secondary	Δ6MWD	6-min walk test	T0, T1	Continuous	MCID reference: 30 m	Increase
Secondary	ΔCAT	COPD Assessment Test	T0, T1	Continuous	MCID reference: 2 points	Decrease
Secondary	ΔmMRC	Modified Medical Research Council dyspnea scale	T0, T1	Ordinal change	Clinically meaningful improvement = ≥1 grade decrease	Decrease
Secondary	ΔResting SpO2	Pulse oximetry	T0, T1	Continuous	Absolute change	Increase
Secondary	ΔBorg dyspnea	Borg scale after 6-min walk test	T0, T1	Continuous	Absolute change	Decrease

MCID, minimal clinically important difference; 6MWD, 6-min walk distance; CAT, COPD assessment test; mMRC, modified Medical Research Council; SpO2, peripheral oxygen saturation.

### 2.4 Data preprocessing, feature selection, modeling strategy, and statistical analysis

A prespecified preprocessing and modeling pipeline was implemented to minimize information leakage and ensure methodological reproducibility. After finalization of the analytic cohort, a single stratified random split was performed using the primary outcome to create training and test sets in a 7:3 ratio. All downstream operations, including missing-data handling, categorical encoding, feature selection, scaling, model fitting, and hyperparameter tuning, were conducted using the training set only. The transformation rules learned from the training data were then applied unchanged to the independent test set. This design prevented information from the test distribution from entering the model-development process. The overall preprocessing, leakage-prevention, model-training, and evaluation workflow is summarized in Table 3.

**Table 3:** Data preprocessing, leakage prevention, model training, and evaluation protocol.

Stage	Procedure	Training-set operation	Test-set operation	Rationale
Cohort construction	Apply inclusion and exclusion criteria and finalize the analytic cohort	Per protocol	Not applied	Ensures consistent eligibility, index definition, and cohort assembly before model development
Data split	Stratified random split (train: test = 7:3) by the primary outcome	Perform once using a fixed random seed	Hold-out set isolated from all model-development procedures	Prevents downstream preprocessing and tuning from using test-set information
Data cleaning	Unit harmonization, range checks, outlier review, and implausible-value handling	Define cleaning rules using the training data only	Apply the same predefined rules without modification	Avoids peeking at the test-set distribution
Missing-data handling	Multiple imputation for numeric predictors and structured handling of missing categorical values	Fit imputation procedures within the training data; embed within cross-validation when applicable	Apply the fitted imputation rules without refitting	Prevents test-set information from influencing imputation
Encoding	Categorical-variable transformation	Fit one-hot or ordinal encoders on training categories according to variable type	Apply training-fitted encoders; unseen categories mapped to Other/Unknown where necessary	Avoids category leakage and preserves reproducibility
Scaling	Algorithm-specific standardization of continuous variables	Fit scaler only for models requiring scale-sensitive input	Apply training-fitted scaler without recalculation	Prevents test statistics from entering feature transformation
Feature selection	LASSO-based predictor screening	Perform in the training data using cross-validation; select penalty by the 1-standard-error rule	Restrict test-set prediction to the training-selected feature subset	Prevents feature-selection bias from the test set
Base-model development	Candidate base-learner fitting	Train candidate learners exclusively in the training data	No training	Prevents overfitting to the independent test set
Hyperparameter tuning	Randomized search with 5-fold cross-validation	Optimize ROC-AUC within the training data only	No tuning	Avoids test-driven model selection
Ensemble stacking	Two-layer stacking architecture	Generate out-of-fold predictions from base learners; fit the logistic-regression meta-learner; refit tuned base learners on the full training set	Generate final predictions once using the trained ensemble	Out-of-fold design reduces optimistic bias caused by information reuse
Class imbalance	Weighted learning strategy	Apply class weights within training folds during model fitting	No reweighting or resampling	Avoids synthetic-data leakage and preserves cleaner evaluation
Final performance evaluation	Discrimination and calibration assessment	Internal cross-validation summaries reported as secondary development information	Primary reporting based on the independent hold-out test set	Provides an unbiased internal estimate of predictive performance
Statistical comparison	Baseline descriptive and between-group analyses	Summarize cohort characteristics and compare Responders versus Non-responders using prespecified tests	Not used for model fitting	Distinguishes descriptive inference from predictive modeling
Explainability	SHAP-based model interpretation	Compute global and local SHAP values after final model fitting	Report global patterns and representative local examples from the test set	Aligns explainability with hold-out model performance

LASSO, least absolute shrinkage and selection operator; ROC-AUC, area under the receiver operating characteristic curve; SHAP, SHapley Additive exPlanations.

Missing numeric predictor values were handled using multiple imputation within the training data. To avoid leakage during model development, imputation procedures were embedded within the resampling framework whenever cross-validation was performed. Categorical variables were encoded using one-hot encoding or clinically meaningful ordinal encoding, depending on variable type and clinical interpretability. Continuous-variable standardization was applied only to algorithms that require scale-sensitive input, such as regularized logistic regression, support vector machine (SVM), and k-nearest neighbors (KNN), whereas tree-based learners were trained on unscaled variables.

Feature selection was performed in the training set using the least absolute shrinkage and selection operator (LASSO). LASSO was selected because it is well suited to moderately high-dimensional clinical datasets with potentially correlated predictors, provides embedded regularization, reduces coefficient instability, and retains stronger clinical interpretability for variable screening than filter-based methods such as ReliefF in the present context. The penalty parameter was selected by cross-validation using the 1-standard-error rule to favor a more parsimonious and stable model. In the present study, 46 candidate predictors entered the feature-screening stage, and 14 predictors were retained in the final LASSO-selected subset for downstream model construction. This number was considered acceptable because feature selection was performed exclusively in the training data, the retained variables were subsequently subjected to regularized or ensemble-based modeling, and final model performance was evaluated on an independent hold-out test set rather than on the same data used for variable screening.

All candidate base learners were prespecified before model fitting. These included regularized logistic regression, random forest, extreme gradient boosting (XGBoost), SVM, and KNN. Logistic regression was included as an interpretable linear benchmark; random forest and XGBoost were selected to capture nonlinear effects and higher-order interactions; SVM was included because of its robustness in moderately high-dimensional classification settings; and KNN was used as a distance-based comparator. Hyperparameters were optimized exclusively in the training set using randomized search with 5-fold cross-validation, with area under the receiver operating characteristic curve (ROC-AUC) as the primary optimization target. To improve reproducibility, the candidate base learners, their roles in the framework, and the representative hyperparameter search space were prespecified in advance. Specifically, logistic regression was tuned with respect to penalty form and regularization strength; random forest was tuned with respect to the number of trees, maximum tree depth, minimum samples required for node split, minimum samples required at terminal nodes, and maximum features; XGBoost was tuned with respect to the number of estimators, learning rate, maximum tree depth, subsample ratio, column-sampling ratio, and gamma; SVM was tuned with respect to kernel type, regularization parameter C, and kernel coefficient gamma; and KNN was tuned with respect to the number of neighbors, weighting scheme, and distance metric. The prespecified candidate base learners and their representative hyperparameter search space are presented in Table 4.

**Table 4:** Prespecified candidate base learners and representative hyperparameter search space.

Model	Role in framework	Representative hyperparameters optimized	Representative search space
Regularized logistic regression	Interpretable linear benchmark and comparator for stacked learning	Penalty type; regularization strength (C)	Penalty: L1 or L2; C: 0.001, 0.01, 0.1, 1, 10, 100
Random forest	Nonlinear ensemble learner for interaction-rich clinical data	Number of trees; maximum depth; minimum samples split; minimum samples leaf; maximum features	n_estimators: 100, 200, 300, 500; max_depth: 3, 5, 7, 10, None; min_samples_split: 2, 5, 10; min_samples_leaf: 1, 2, 4; max_features: sqrt, log2, None
XGBoost	Gradient-boosting learner for nonlinear effects and complex feature interactions	Number of estimators; learning rate; maximum depth; subsample; colsample_bytree; gamma	n_estimators: 100, 200, 300, 500; learning_rate: 0.01, 0.05, 0.1, 0.2; max_depth: 3, 4, 5, 6; subsample: 0.6, 0.8, 1.0; colsample_bytree: 0.6, 0.8, 1.0; gamma: 0, 0.1, 0.3, 0.5
Support vector machine (SVM)	Margin-based classifier for moderately high-dimensional classification	Kernel type; regularization parameter C; kernel coefficient gamma	Kernel: linear, rbf; C: 0.1, 1, 10, 100; gamma: scale, auto, 0.01, 0.1, 1
k-Nearest neighbors (KNN)	Distance-based comparator sensitive to local neighborhood structure	Number of neighbors; weighting scheme; distance metric	n_neighbors: 3, 5, 7, 9, 11, 15; weights: uniform, distance; metric: euclidean, manhattan, minkowski
Logistic-regression meta-learner	Second-layer learner for stacking aggregation	Penalty type; regularization strength (C)	Penalty: L2; C: 0.01, 0.1, 1, 10

SVM, support vector machine; KNN, *k*-nearest neighbors; XGBoost, extreme gradient boosting.

The final ensemble framework used a two-layer stacking strategy. In the first layer, each base learner generated out-of-fold predictions through cross-validation within the training set. These out-of-fold predictions were then used as inputs to a second-layer meta-learner. Logistic regression was selected as the meta-learner to preserve transparency and provide stable aggregation of base-model predictions. After tuning was completed, each base learner was refitted on the full training set using the optimized hyperparameters, and the trained meta-learner was subsequently applied to generate final predictions for the independent test set. This out-of-fold design reduced optimistic bias associated with information reuse and improved ensemble stability.

Class imbalance was handled by class-weighted learning within the training process rather than by synthetic resampling. This approach was chosen to reduce the risk of introducing artificial structure into the data and to preserve a cleaner evaluation pipeline in the presence of an independent hold-out test set.

Model performance was evaluated primarily in the independent test set. The main performance metrics included area under the receiver operating characteristic curve (ROC-AUC), area under the precision-recall curve (PR-AUC), sensitivity, specificity, F1 score, and Brier score, together with calibration assessment based on calibration curves, calibration slope, and calibration intercept. Ninety-five percent confidence intervals were estimated by bootstrap resampling of the test set. Internal cross-validation summaries from the training data were reported only as secondary model-development information and were not treated as the principal basis for performance claims.

Descriptive statistics were used to summarize baseline characteristics. Continuous variables are presented as mean ± standard deviation or median with interquartile range, depending on distributional properties, whereas categorical variables are reported as counts and percentages. Comparisons between Responders and Non-responders were performed using the Student t test or Mann–Whitney U test for continuous variables and the chi-square test or Fisher exact test for categorical variables, as appropriate. These inferential analyses were used to describe cohort characteristics and group differences rather than to substitute for model-based predictive evaluation.

Model explainability was assessed using SHapley Additive exPlanations (SHAP). Global SHAP importance was used to rank influential predictors across the model, whereas local SHAP values were used to illustrate patient-level prediction mechanisms in representative cases.

### 2.5 Dataset split and training strategy

After cohort construction, eligibility confirmation, and initial data harmonization, the final analytic dataset was divided into training and test sets using a stratified random split at a ratio of 7:3 according to the primary outcome. The training set was used for feature preprocessing, feature selection, base-learner development, hyperparameter tuning, and meta-learner construction, whereas the test set was reserved exclusively for independent performance evaluation and *post hoc* explainability analysis.

To prevent information leakage, all preprocessing procedures were fitted in the training set first and then applied unchanged to the test set. These procedures included data cleaning, missing-data handling, categorical encoding, and model-specific scaling when required. Cross-validation was implemented only within the training set, and the final ensemble was constructed through a stacking framework based on out-of-fold predictions. The overall preprocessing, leakage-prevention, and evaluation workflow is summarized in Table 3, whereas the candidate base learners and their representative hyperparameter search space are presented in Table 4.

### 2.6 Feature selection using LASSO

To reduce redundancy among candidate predictors and improve model parsimony, feature selection was performed within the training dataset using the least absolute shrinkage and selection operator (LASSO). Cross-validation was used to identify the penalty parameter, and the 1-standard-error rule was adopted to favor a more stable and parsimonious subset of predictors.

LASSO was selected because it combines variable screening with embedded regularization and is well suited to clinical datasets containing correlated and moderately high-dimensional predictors. In the present study, 46 candidate predictors entered the feature-screening stage, and 14 predictors were retained in the final subset for downstream model development. This approach was considered more appropriate than filter-based alternatives because it preserved a direct connection between feature selection and predictive modeling while maintaining clearer clinical interpretability. Feature selection was conducted exclusively within the training set and was not informed by any data from the test set.

### 2.7 Model development

Multiple candidate base learners were developed in the training dataset with concurrent hyperparameter optimization. These candidate models included regularized logistic regression, random forest, extreme gradient boosting (XGBoost), support vector machine (SVM), and k-nearest neighbors (KNN). Each model was selected to contribute a distinct inductive bias, thereby enabling the ensemble framework to capture linear effects, nonlinear relationships, and higher-order interactions among predictors.

A stacked ensemble framework was then used to construct the final predictive system. During cross-validation within the training set, each base learner generated out-of-fold predictions, which were subsequently used as inputs to the second-layer meta-learner. The meta-learner was specified as logistic regression to preserve aggregation stability and improve interpretability of the final ensemble output. After optimization and model fitting were completed in the training set, the finalized stacked model was applied to the independent test set to generate final predictions and support subsequent explainability analysis.

### 2.8 Performance metrics and subgroup analysis plan

Model discrimination was evaluated primarily using the area under the receiver operating characteristic curve (ROC-AUC), together with the area under the precision-recall curve (PR-AUC), sensitivity, specificity, and F1 score. Calibration performance was assessed using calibration curves, the Brier score, calibration slope, and calibration intercept. All primary performance indicators were calculated in the independent test set, and 95% confidence intervals were estimated by bootstrap resampling.

Because the present study used internal rather than true external validation, subgroup analyses were treated as exploratory rather than confirmatory. Model performance was additionally examined across selected clinically relevant strata, including diagnosis group, baseline functional capacity, baseline symptom burden, inflammatory status, rehabilitation adherence, TCM co-intervention intensity, age, sex, care setting, and participating center, in order to assess whether predictive behavior remained broadly consistent across different clinical contexts. Subgroup sample sizes and key performance metrics were summarized to explore potential heterogeneity and to delineate the practical boundaries of model applicability.

## 3 Results

### 3.1 Cohort characteristics and dataset description

A total of 1,483 patients with respiratory diseases who met the prespecified eligibility criteria were included in the final analytic cohort. According to the predefined stratified random split, 1,038 patients were assigned to the training set and 445 to the independent test set. Baseline demographic and clinical characteristics, disease severity, physiological indicators, baseline functional status, and outcome distribution across the two subsets are presented in Table 5. Overall, the distributions of major variables were broadly comparable between the training and test sets, indicating that the stratified split preserved a similar cohort structure for model development and hold-out evaluation.

**Table 5:** Baseline characteristics and outcome distribution of participants in the training and test sets.

Characteristics	Training: non-responders n=388	Training: responders n=650	Training *P*	Testing: non-responders n=165	Testing: responders n=280	Testing *P*
Participants, *n*	388	650		165	280	
Age (years)	63.430 ± 13.812	56.407 ± 12.546	<0.001	61.804 ± 11.886	55.266 ± 11.104	<0.001
BMI kg/m2	25.618 ± 4.180	24.142 ± 3.633	<0.001	25.058 ± 3.987	24.216 ± 3.574	0.026
CAT score, baseline	21.997 ± 7.066	18.154 ± 6.075	<0.001	21.451 ± 7.203	18.769 ± 6.420	<0.001
6MWD, baseline (m)	325.423 ± 84.791	375.129 ± 87.623	<0.001	329.520 ± 89.695	375.338 ± 89.273	<0.001
Resting SpO2, baseline (%)	94.825 ± 2.650	96.018 ± 2.015	<0.001	94.691 ± 2.505	96.158 ± 1.929	<0.001
Respiratory rate, baseline (breaths/min)	19.438 ± 3.119	18.225 ± 2.675	<0.001	19.382 ± 3.077	18.306 ± 2.741	0.001
Heart rate, baseline (bpm)	82.309 ± 13.089	78.002 ± 12.219	<0.001	82.718 ± 13.311	78.609 ± 12.403	0.004
NLR, baseline	3.284 [2.475, 4.460]	2.666 [2.050, 3.624]	<0.001	3.172 [2.329, 4.292]	2.752 [2.098, 3.785]	0.003
Charlson Comorbidity Index (CCI)	2.000 [1.000, 3.000]	1.000 [1.000, 2.000]	<0.001	2.000 [1.000, 3.000]	1.000 [1.000, 2.000]	<0.001
mMRC grade, baseline	2.000 [2.000, 3.000]	2.000 [1.000, 3.000]	<0.001	2.000 [2.000, 3.000]	2.000 [1.000, 3.000]	0.002
Male sex, *n* (%)	228 (58.763%)	351 (54.000%)	0.135	98 (59.394%)	147 (52.500%)	0.159
Smoking status, *n* (%)			0.003			0.118
Never	166 (42.784%)	353 (54.308%)		79 (47.879%)	142 (50.714%)	
Former	123 (31.701%)	190 (29.231%)		52 (31.515%)	84 (30.000%)	
Current	99 (25.515%)	107 (16.462%)		34 (20.606%)	54 (19.286%)	
Primary diagnosis, *n* (%)			<0.001			0.002
COPD	220 (56.701%)	268 (41.231%)		86 (52.121%)	117 (41.786%)	
Post-viral	104 (26.804%)	236 (36.308%)		50 (30.303%)	103 (36.786%)	
Asthma	38 (9.794%)	104 (16.000%)		15 (9.091%)	40 (14.286%)	
Other	26 (6.701%)	42 (6.462%)		14 (8.485%)	20 (7.143%)	
Inpatient setting, *n* (%)	175 (45.103%)	202 (31.077%)	<0.001	77 (46.667%)	88 (31.429%)	0.002
Hospital site, *n* (%)			0.879			0.689
Site 1	107 (27.577%)	179 (27.538%)		45 (27.273%)	87 (31.071%)	
Site 2	110 (28.351%)	172 (26.462%)		39 (23.636%)	62 (22.143%)	
Site 3	94 (24.227%)	170 (26.154%)		50 (30.303%)	73 (26.071%)	
Site 4	77 (19.845%)	129 (19.846%)		31 (18.788%)	58 (20.714%)	

Data are presented as mean ± standard deviation, median [interquartile range], or *n* (%), as appropriate. *P* values were calculated using the student *t* test or Mann–Whitney U test for continuous variables and the chi-square test or Fisher exact test for categorical variables, as appropriate.

BMI, body mass index; CAT, COPD Assessment Test; 6MWD, 6-min walk distance; SpO2, peripheral oxygen saturation; NLR, neutrophil-to-lymphocyte ratio; CCI, Charlson Comorbidity Index; mMRC, modified Medical Research Council dyspnea scale; COPD, chronic obstructive pulmonary disease.

Clear baseline differences were observed between Responders and Non-responders in both the training and test sets. Compared with Responders, Non-responders tended to be older and to show a less favorable baseline clinical profile, including higher symptom burden, greater dyspnea severity, lower baseline exercise tolerance, lower resting oxygen saturation, higher respiratory and heart rates, greater inflammatory burden, and higher comorbidity scores. Several of these between-group patterns were directionally consistent across the training and test sets, supporting their clinical relevance for subsequent predictive modeling.

With respect to categorical variables, the Non-responder group showed a higher proportion of inpatient cases in both subsets, suggesting that care setting may reflect differences in baseline severity or rehabilitation context. By contrast, the distribution of hospital sites remained generally balanced across outcome groups in both the training and test sets, indicating limited center-related imbalance at the cohort level. Smoking status and primary diagnosis also differed between outcome groups, particularly in the training set, further supporting the inclusion of these variables in the candidate predictor framework.

**Figure 2:**
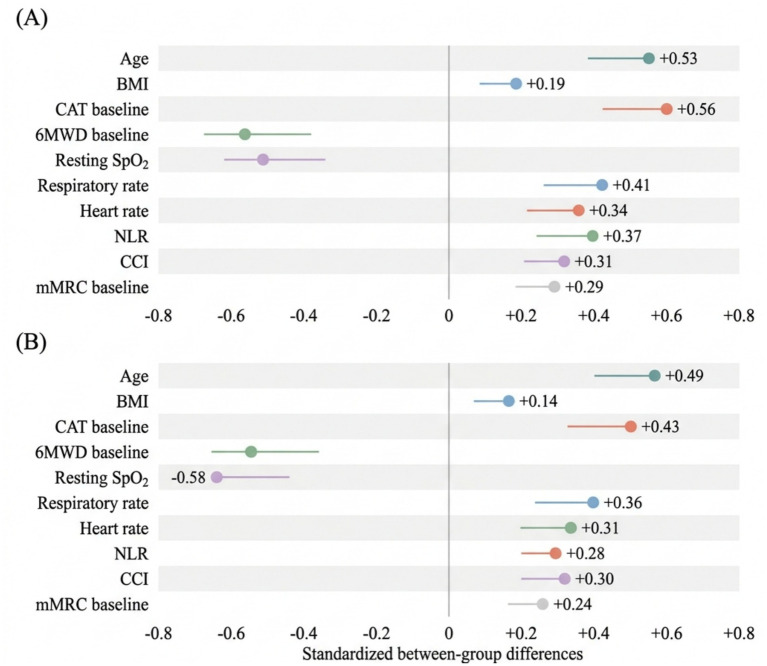
Visual comparison of baseline differences between responders and non-responders in the training and test sets. **(A)** Standardized between-group differences for key continuous baseline variables in the training set, displayed as horizontal dot-and-error plots centered on a zero reference line. **(B)** Corresponding standardized between-group differences for the same baseline variables in the independent test set, using the same variable order, axis scale, and visual style for direct comparison.

To improve the readability of the baseline comparisons, the principal continuous variables are additionally visualized in Figure 2. As shown in Figure 2, the overall direction and relative magnitude of the between-group differences were broadly similar in the training and test sets, further supporting the internal consistency of the dataset.

Panel A shows the standardized between-group differences for key continuous baseline variables in the training set, and Panel B shows the corresponding differences in the independent test set. Positive values indicate higher levels in Non-responders, whereas negative values indicate higher levels in Responders. Across both subsets, Non-responders generally showed older age, higher baseline symptom burden, lower baseline 6-min walk distance, less favorable oxygenation, and higher inflammatory burden. This figure complements the numerical comparisons shown in Table 5.

### 3.2 Feature selection results

**Figure 3:**
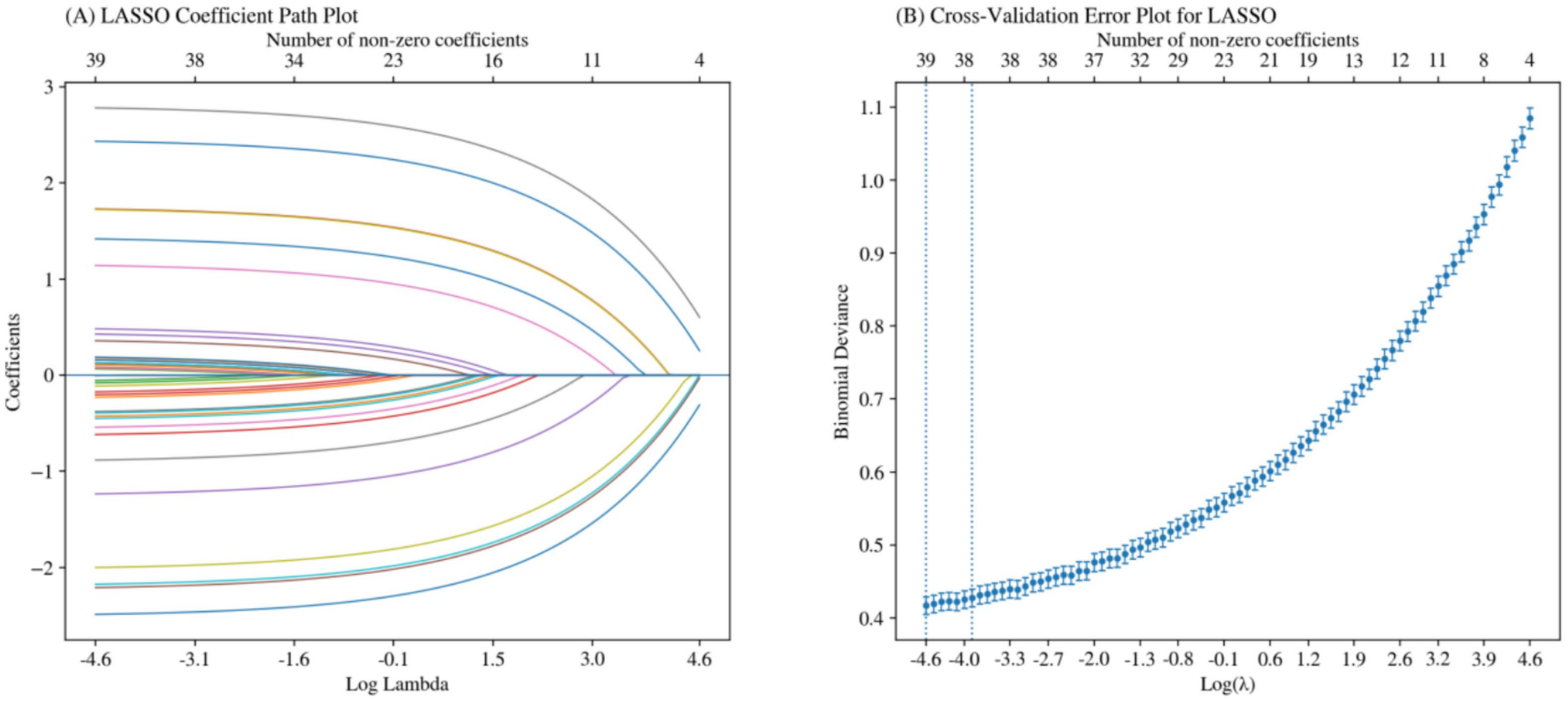
LASSO-based feature selection in the training set: **(A)** Coefficient paths and **(B)** cross-validated error curve for predictor reduction.

LASSO regression was performed in the training set to reduce predictor redundancy and improve model parsimony before downstream model development. As the penalty parameter *λ* increased, regression coefficients progressively shrank toward zero, and less stable or less informative variables were excluded from the model. The coefficient trajectories showed orderly shrinkage behavior, indicating stable regularization of the retained features (Figure 3A).

Cross-validation showed that model error reached its minimum within a narrow range of λ values and remained acceptably stable around the optimal solution (Figure 3B). According to the prespecified 1-standard-error rule, 46 candidate predictors entered the feature-screening stage and 14 predictors with non-zero coefficients were retained for subsequent model construction. These retained variables were then carried forward into the candidate base learners and the stacked ensemble framework for training and independent evaluation.

### 3.3 Model performance in the independent test set

**Figure 4:**
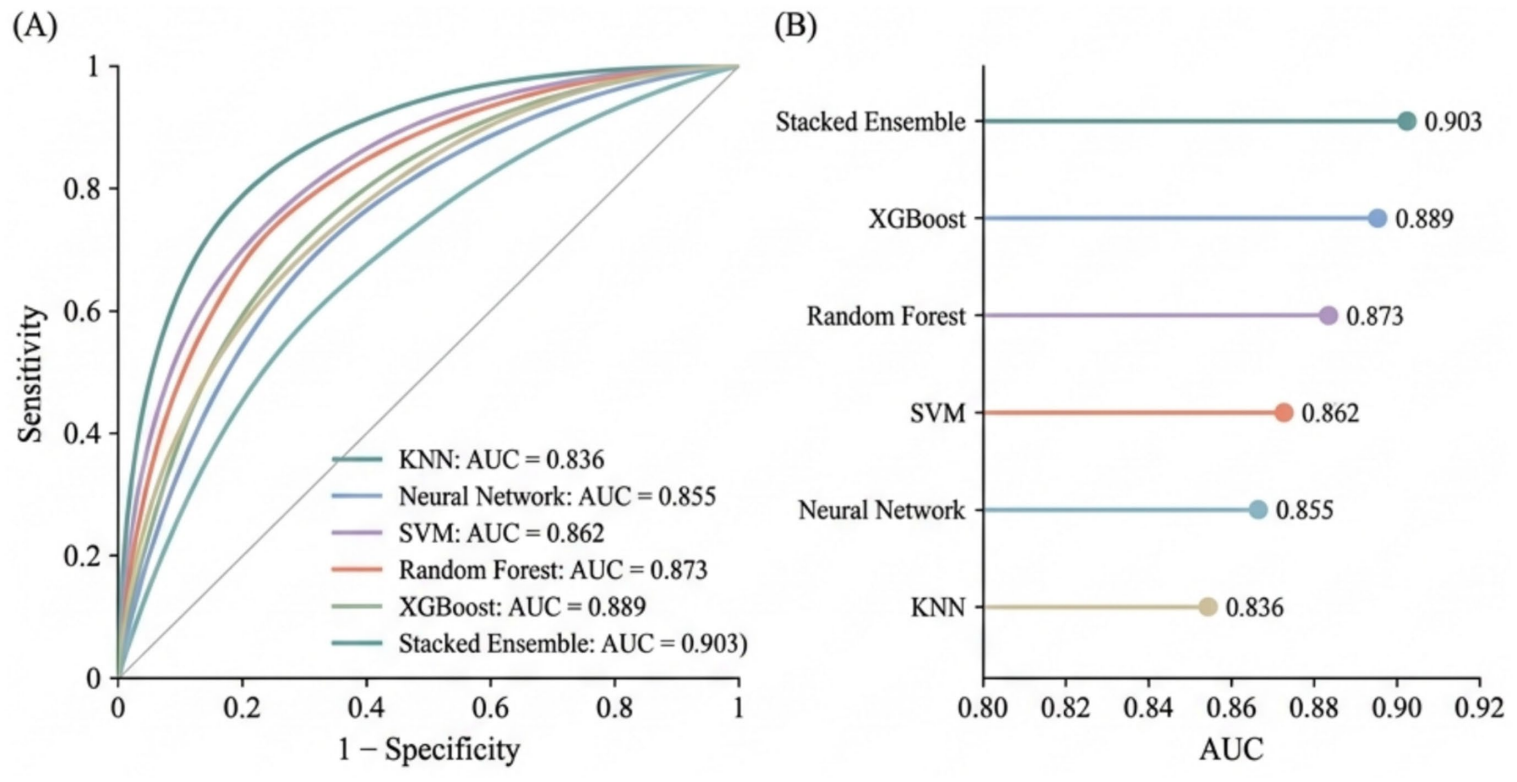
Comparative receiver operating characteristic curves for the candidate machine-learning models and the stacked ensemble model in the independent test set. **(A)** Comparative ROC curves of KNN, neural network, random forest, SVM, XGBoost, and the stacked ensemble model in the independent test set, with AUC values shown in the legend. **(B)** Ranked AUC summary plot of the six candidate models, using color-matched bars or dots to correspond with the ROC curves in Panel A.

All candidate models demonstrated meaningful discriminative ability in the independent test set, although performance varied across algorithms. Overall, the tree-based models, particularly random forest and XGBoost, outperformed the distance-based and margin-based approaches, whereas the stacked ensemble model achieved the best overall discrimination among all evaluated methods. As shown in Figure 4, the ensemble model consistently maintained the most favorable receiver operating characteristic profile during hold-out testing.

Model calibration was also examined in parallel with discrimination. As summarized in Table 6, the stacked ensemble model achieved the highest ROC-AUC, PR-AUC, accuracy, sensitivity, specificity, and F1 score, while also showing the lowest Brier score. Its calibration slope was closest to 1 and its calibration intercept was closest to 0, indicating better agreement between predicted probabilities and observed outcome frequencies than the individual models. By contrast, several single-model approaches showed mild deviations in calibration despite acceptable discrimination. Taken together, these findings indicate that the stacked ensemble model provided the most balanced performance across discrimination and calibration dimensions and was therefore selected as the final model for subsequent interpretability and exploratory subgroup analyses.

**Table 6:** Performance of individual models and the ensemble model on the independent test set.

Model	AUC	PR-AUC	Accuracy	Sensitivity	Specificity	F1-score	Brier score	Calibration slope	Calibration intercept	Hosmer–Lemeshow *p*
KNN	0.836	0.889	0.806	0.862	0.667	0.857	0.162	0.874	0.071	0.048
NN	0.855	0.903	0.818	0.871	0.689	0.866	0.154	0.916	0.052	0.093
RF	0.873	0.915	0.832	0.879	0.716	0.876	0.146	0.944	0.031	0.168
SVM	0.862	0.909	0.825	0.883	0.694	0.872	0.15	0.932	0.041	0.121
XGBoost	0.889	0.927	0.845	0.892	0.742	0.885	0.139	0.967	0.018	0.237
Ensemble (stacking)	0.903	0.938	0.857	0.906	0.761	0.899	0.131	0.986	−0.009	0.412

**Figure 5:**
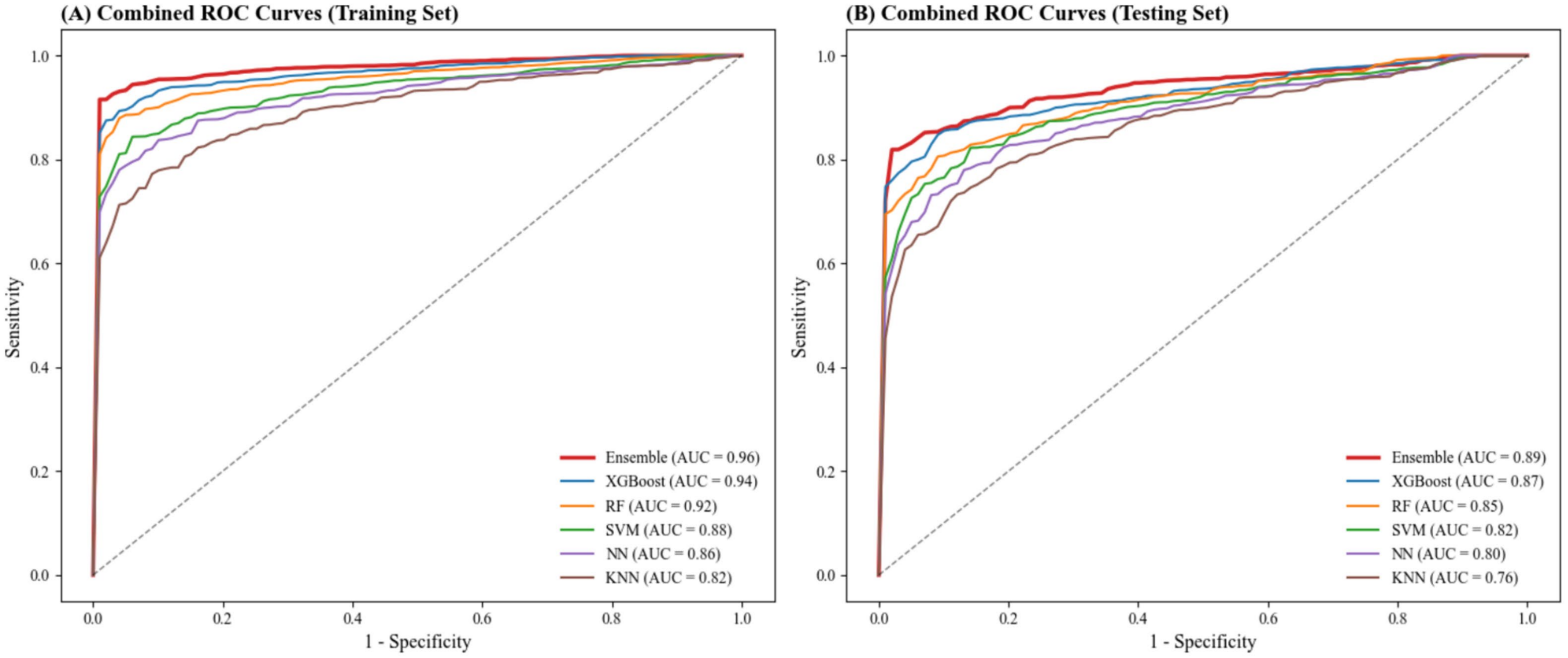
Comparative performance of candidate machine-learning models in the independent test set. **(A)** Summarizes discrimination metrics, including ROC-AUC and PR-AUC, for each candidate model. **(B)** Summarizes calibration-related performance, including Brier score and calibration slope. The stacked ensemble model showed the most favorable overall balance between discrimination and calibration, with the highest ROC-AUC and PR-AUC, the lowest Brier score, and a calibration slope closest to 1. This figure complements Table 6 and facilitates cross-model comparison beyond the receiver operating characteristic curves shown in Figure 4.

For a more intuitive comparison of model behavior beyond the receiver operating characteristic curves, the principal discrimination and calibration metrics are additionally visualized in Figure 5.

### 3.4 Global interpretability: key predictors driving outcome evaluation

**Figure 6:**
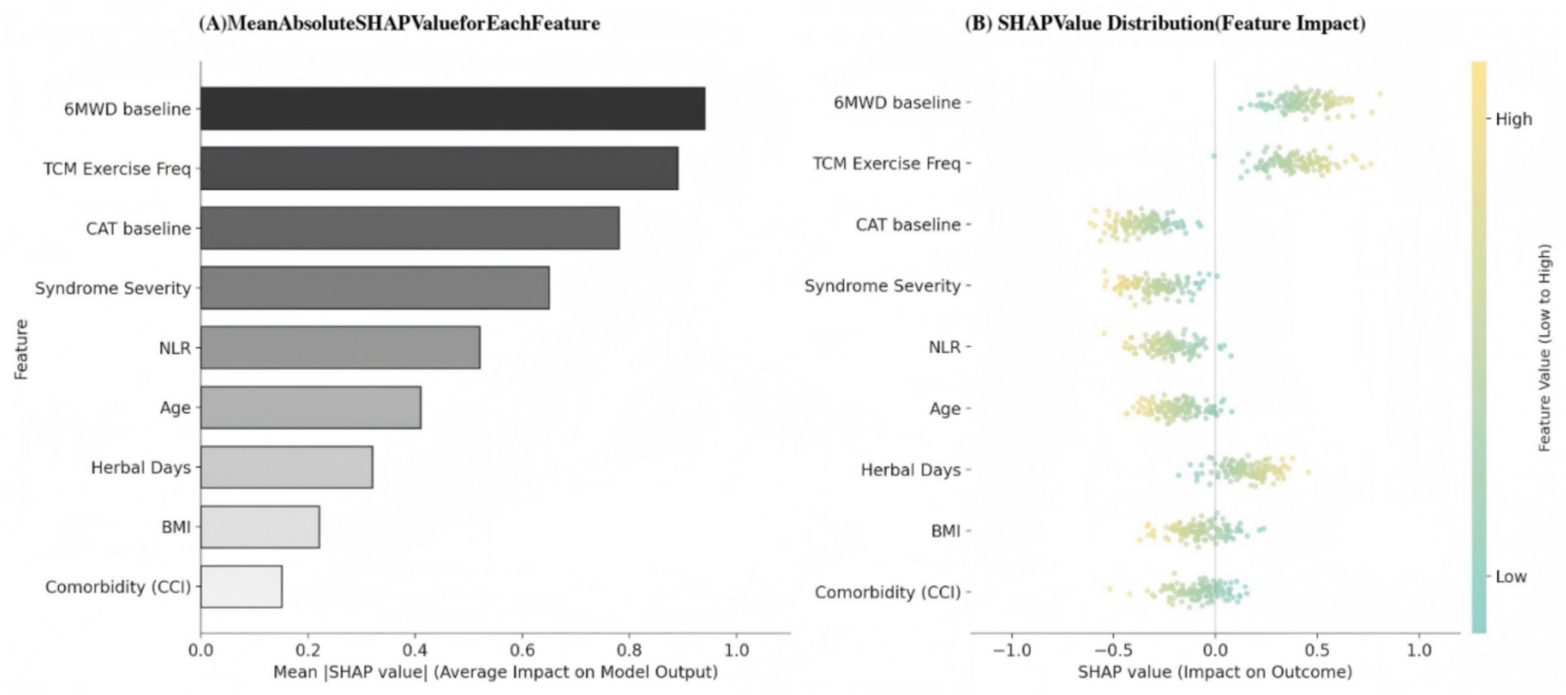
Global SHAP results for the final ensemble model: **(A)** Mean absolute SHAP importance ranking and **(B)** SHAP summary distribution plot.

To enhance interpretability in the context of TCM-integrated respiratory rehabilitation, global SHAP analysis was performed on the final stacked ensemble model. By quantifying the marginal contribution of each predictor to the model-estimated probability of rehabilitation response, the analysis identified the variables that most strongly influenced prediction. The global importance ranking is shown in Figure 6A, and the corresponding SHAP summary distribution is shown in Figure 6B.

The results indicated that baseline measures of functional capacity and symptom burden, particularly baseline 6-min walk distance (6MWD) and baseline COPD Assessment Test (CAT) score, ranked among the most influential predictors in terms of mean absolute SHAP value. These findings indicate that baseline exercise tolerance and symptom severity were strongly represented in the final model output. At the same time, TCM-related variables also made stable contributions, with TCM exercise frequency and Herbal Days ranking prominently in the importance hierarchy. This pattern suggests that indicators of TCM intervention intensity were consistently captured by the model in relation to rehabilitation outcome classification.

According to the SHAP summary distribution, higher baseline 6MWD and greater TCM exercise frequency were more frequently associated with positive SHAP values, indicating that larger feature magnitudes tended to correspond to a higher predicted probability of response. Conversely, higher baseline CAT score, greater syndrome severity, and higher neutrophil-to-lymphocyte ratio (NLR) were more often linked to negative SHAP values, indicating a lower predicted likelihood of rehabilitation response. Age, body mass index (BMI), and comorbidity burden generally showed smaller SHAP magnitudes but followed similar directional tendencies.

### 3.5 Nonlinear effects: SHAP dependence analysis

To further examine nonlinear associations and possible threshold-like patterns among major continuous predictors and the model output, SHAP dependence analyses were conducted using the final stacked ensemble model. These plots illustrate how variation in feature values was associated with changes in marginal contribution to the predicted probability of rehabilitation response while also visualizing potential interaction structure with rehabilitation training adherence.

**Figure 7:**
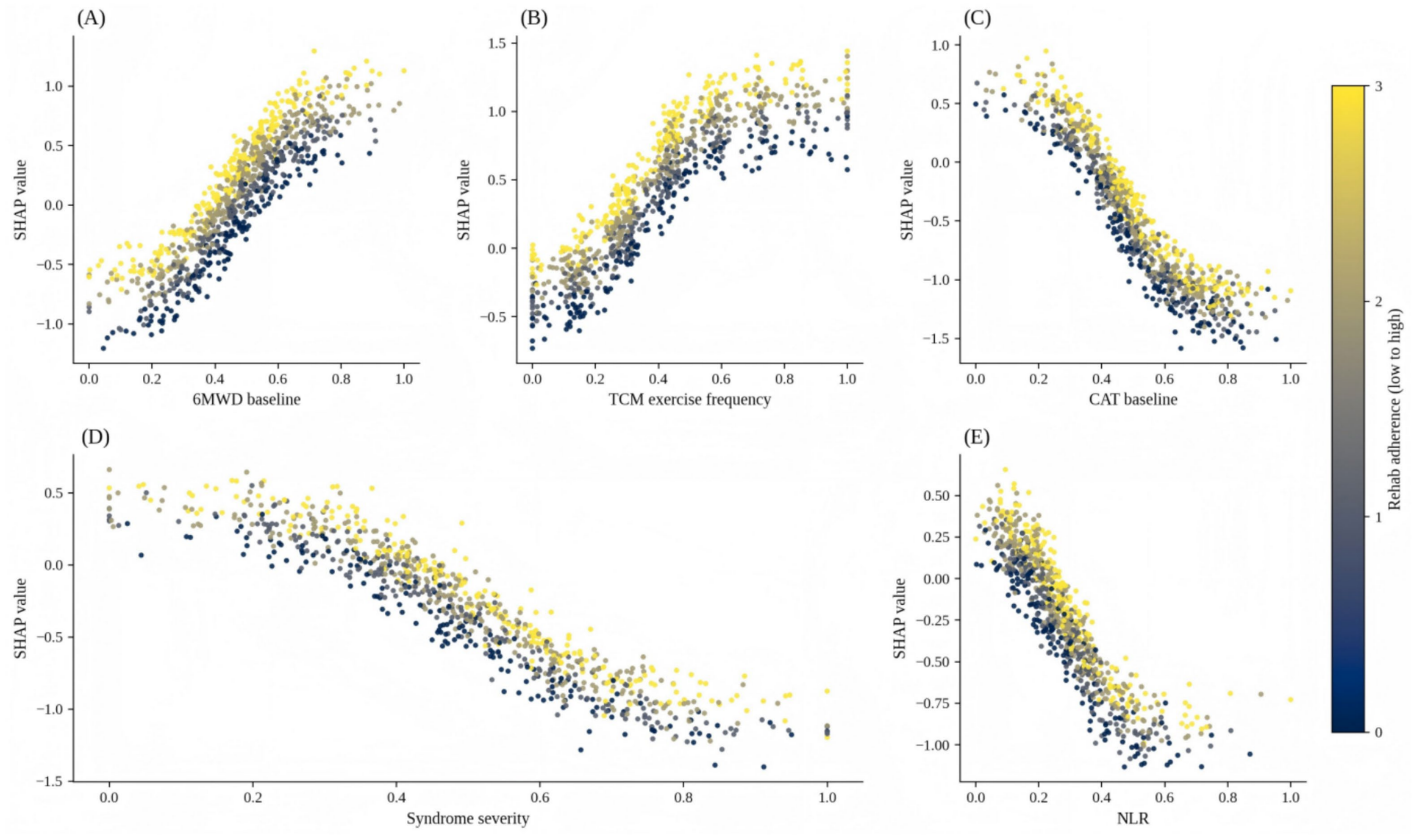
SHAP dependence plots for key continuous predictors in the final ensemble model, showing nonlinear effects and potential threshold patterns.

The SHAP values for baseline 6MWD showed an overall increasing trend as feature magnitude increased, although the rate of increase appeared to attenuate in the mid-to-high range. Within the final model, higher baseline exercise capacity therefore corresponded to a greater predicted probability of response, with a visible flattening pattern at higher levels. TCM exercise frequency also demonstrated a positive nonlinear association, with larger marginal gains in the low-to-moderate range and relatively stable contributions at higher values, as shown in Figures 7A,B.

Baseline CAT score showed a consistently negative nonlinear association with the predicted outcome, particularly within the moderate-to-high range where SHAP values became more strongly negative. Syndrome severity score followed a similar downward pattern, with higher values corresponding to lower predicted probabilities of response and a denser concentration of negative contributions among patients with more severe baseline syndrome burden, as shown in Figures 7C,D. In addition, NLR demonstrated a negative relationship with SHAP values, with more pronounced declines observed in the lower-to-middle portion of the feature distribution, as shown in Figure 7E.

Across several dependence plots, the color gradient representing rehabilitation training adherence suggested that higher adherence tended to align with more favorable SHAP contributions or with attenuation of negative SHAP values. Within the final model, adherence was therefore captured as a potential interacting factor in the relationship between baseline characteristics and predicted rehabilitation response.

### 3.6 Individual-level explanations for clinical interpretability

**Figure 8:**
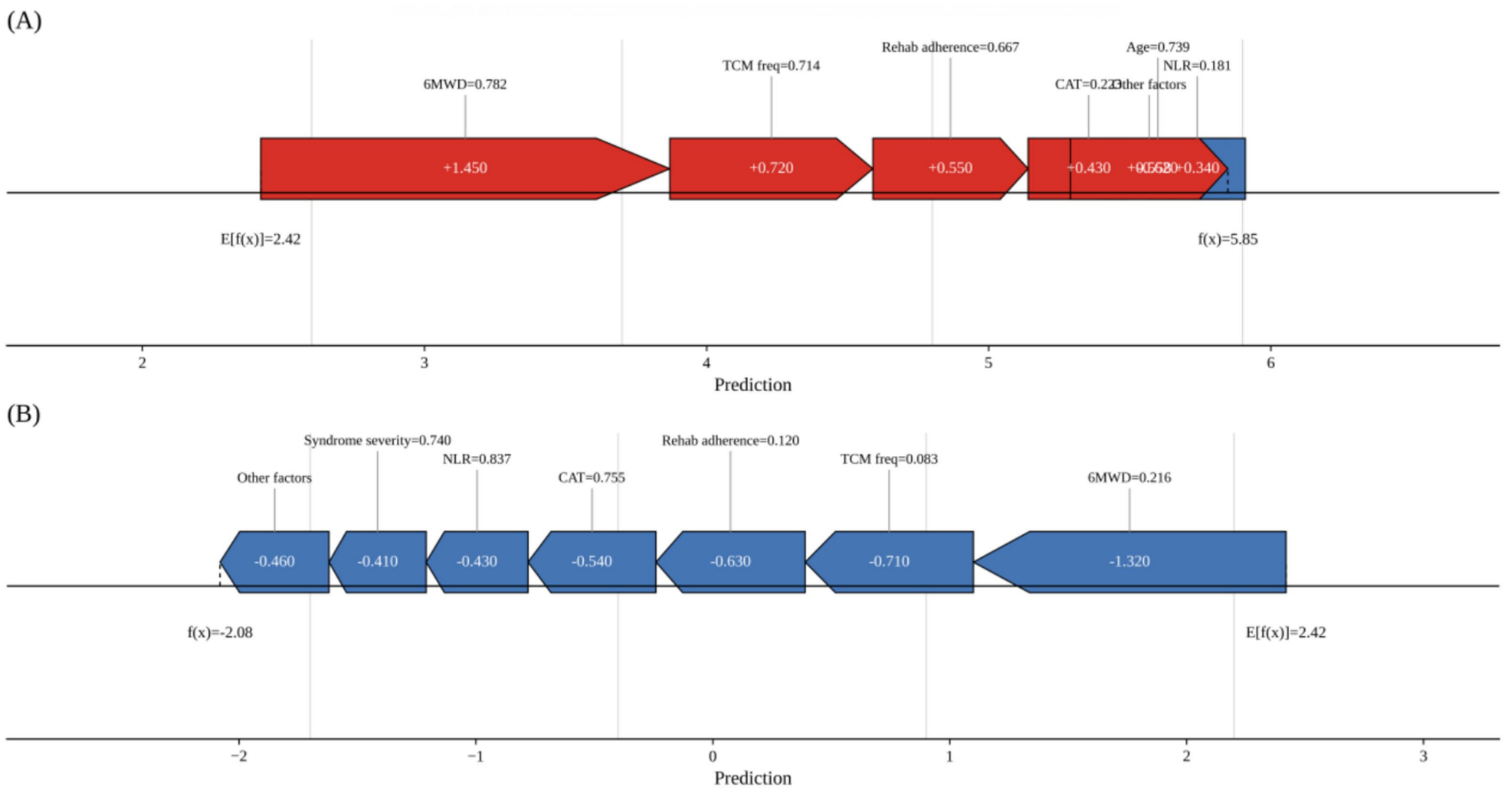
Patient-level SHAP force plots for representative cases classified as **(A)** responder and **(B)** non-responder.

To improve the clinical interpretability of the final model, individual-level SHAP force-plot analyses were further used to illustrate how each patient’s predicted probability was generated through the combined contributions of positive and negative features. This case-level visualization framework converts the model output into a traceable attribution pattern and clarifies how multiple predictors jointly shape the final classification. Representative examples of a correctly classified Responder and a correctly classified Non-responder are shown in Figure 8.

In the representative Responder case, baseline functional indicators and intervention-related variables, including higher baseline 6-min walk distance (6MWD), greater frequency of TCM exercise therapy, and stronger rehabilitation adherence, contributed positively and shifted the prediction toward the Responder class. Although several variables, such as residual symptom burden and inflammatory markers, still exerted negative contributions, the cumulative SHAP attribution remained predominantly favorable, resulting in final classification as a Responder (Figure 8A).

In the representative Non-responder case, disease burden, inflammatory indicators such as neutrophil-to-lymphocyte ratio (NLR), and symptom burden reflected by baseline COPD Assessment Test (CAT) score showed stronger negative SHAP contributions, shifting the prediction toward the Non-responder class. Although some intervention-related variables still contributed positively, their magnitude was comparatively smaller, and the overall attribution pattern remained unfavorable (Figure 8B).

Taken together, these patient-level explanations show that the model output was not driven by any single variable in isolation, but rather by the integrated influence of baseline function, symptom burden, inflammatory status, and intervention-related features.

### 3.7 Subgroup performance and heterogeneity

**Figure 9:**
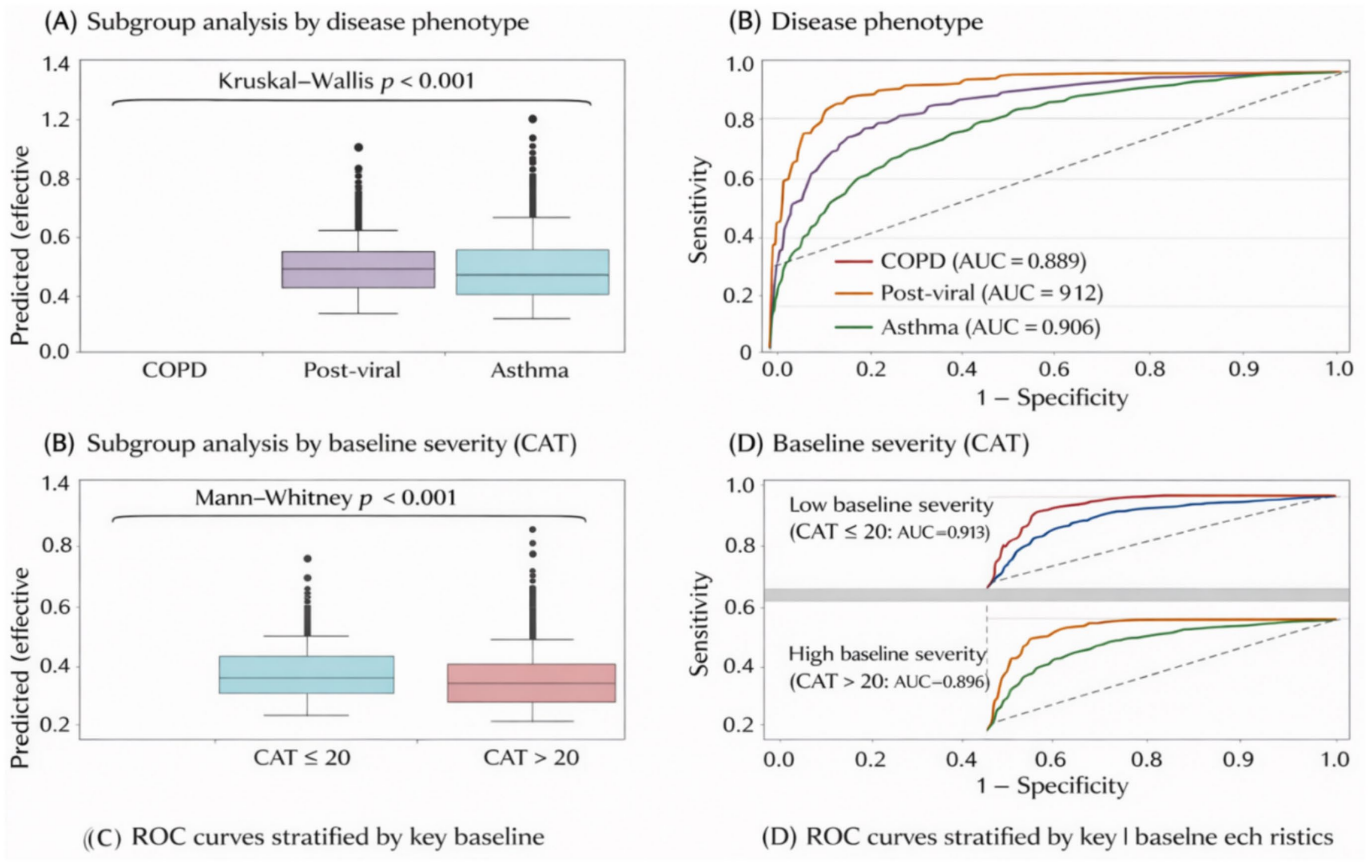
Forest plot of subgroup-specific ROC-AUC estimates for the final ensemble model in the independent test set.

**Figure 10:**
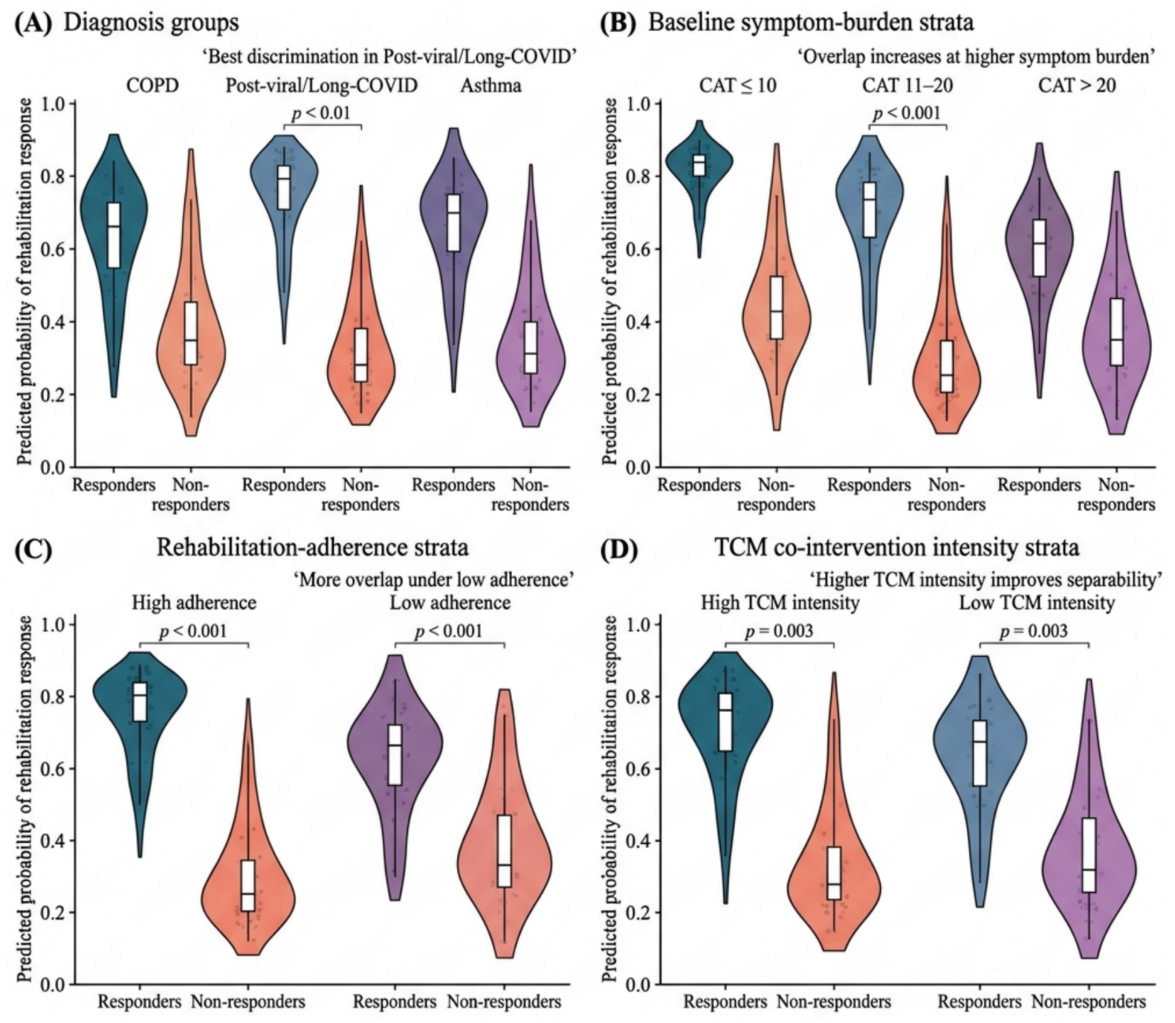
Distribution of predicted probabilities of rehabilitation response across key clinical subgroups in the independent test set. **(A)** Violin–boxplot distributions of predicted rehabilitation-response probabilities in responders and non-responders across diagnosis groups (COPD, Post-viral/Long-COVID, Asthma). **(B)** Violin–boxplot distributions across baseline symptom-burden strata (CAT ≤10, CAT 11–20, CAT >20). **(C)** Violin–boxplot distributions across rehabilitation-adherence strata (high adherence vs. low adherence). **(D)** Violin–boxplot distributions across TCM co-intervention intensity strata (high TCM intensity vs. low TCM intensity).

To further examine the robustness of the final stacked ensemble model in clinically relevant settings, exploratory subgroup analyses were performed using prespecified stratification variables in the independent test set. Subgroup-specific performance estimates are summarized numerically in Table 7 and visually in Figure 9, whereas the distributions of predicted probabilities across selected clinical strata are shown in Figure 10.

**Table 7:** Exploratory subgroup performance summary for the final ensemble model in the independent test set.

Predefined stratum	Stratum level	n	Event rate (Responders, %)	ROC-AUC	Brier score	Sensitivity	Specificity	Key heterogeneity/interaction notes
Age	<60 years	238	67.227	0.915	0.124	0.912	0.764	Best discrimination; gain mainly driven by functional baseline and adherence features
	≥60 years	207	58.454	0.889	0.142	0.896	0.741	Slightly reduced discrimination; comorbidity and inflammation features contributed more
Sex	Male	245	60.000	0.901	0.133	0.905	0.752	Similar performance to the overall model; no obvious interaction pattern
	Female	200	66.500	0.907	0.129	0.907	0.760	Stable calibration; rehabilitation adherence remained an important contributor
Diagnosis	COPD	203	57.635	0.892	0.146	0.894	0.739	Lower discrimination than other diagnosis groups; NLR and syndrome severity showed stronger negative influence
	Post-viral / long-COVID	153	67.320	0.919	0.121	0.918	0.768	Highest discrimination; TCM exercise frequency showed larger marginal benefit
	Asthma	55	72.727	0.906	0.127	0.914	0.744	Good performance; symptom burden remained a dominant driver
	Other	34	58.824	0.881	0.149	0.882	0.721	Smaller subgroup; wider uncertainty and less stable calibration
Baseline functional capacity (6MWD)	<300 m	118	50.847	0.873	0.164	0.882	0.701	Most challenging subgroup; uncertainty increased at lower functional baseline
	300–450 m	227	63.877	0.912	0.128	0.909	0.762	Strong performance; balanced sensitivity and specificity
	>450 m	100	73.000	0.905	0.112	0.924	0.736	Possible ceiling effect; discrimination slightly attenuated because of high event rate
Baseline symptom burden (CAT)	<10	61	72.131	0.886	0.118	0.931	0.688	Lower discrimination likely related to limited variability and high event rate
	10–20	187	67.914	0.913	0.126	0.910	0.765	Stable performance; major drivers remained 6MWD and rehabilitation adherence
	>20	197	54.315	0.898	0.149	0.891	0.748	Greater heterogeneity; CAT and syndrome severity showed stronger negative influence
Inflammation (NLR)	<3.0	244	68.033	0.914	0.123	0.913	0.763	Better calibration; functional and intervention-intensity features dominated
	≥3.0	201	56.716	0.889	0.145	0.894	0.741	Reduced discrimination; inflammation and comorbidity contributed more
Rehabilitation adherence level	High (≥2.5/3)	214	72.430	0.916	0.118	0.922	0.752	Best performance; TCM and rehabilitation-intensity features showed clearer positive effects
	Low <2.5/3	231	54.113	0.887	0.151	0.882	0.744	Discrimination declined and uncertainty widened; adherence appeared to be a major source of heterogeneity
TCM co-intervention intensity	High (Herbal days ≥14 and/or acupuncture sessions ≥8)	186	69.892	0.912	0.125	0.915	0.758	Strong discrimination; TCM exercise frequency and herbal duration contributed more
	Low (below threshold)	259	57.143	0.895	0.142	0.895	0.751	Slightly weaker discrimination; outcomes relied more on baseline function and rehabilitation dose
Hospital site	Site 1	132	65.909	0.907	0.130	0.904	0.763	No major site interaction; performance remained consistent
	Site 2	101	61.386	0.899	0.138	0.895	0.750	Stable
	Site 3	123	59.350	0.904	0.133	0.906	0.748	Stable
	Site 4	89	65.169	0.898	0.137	0.899	0.747	Stable

ROC-AUC, area under the receiver operating characteristic curve; 6MWD, 6-min walk distance; CAT, COPD assessment test; NLR, neutrophil-to-lymphocyte ratio; TCM, traditional Chinese medicine; COPD, chronic obstructive pulmonary disease.

Overall, the model maintained acceptable discriminative performance across the major diagnosis groups, including chronic obstructive pulmonary disease (COPD), post-viral respiratory syndromes, and asthma, as well as across strata defined by baseline symptom burden, functional capacity, inflammatory status, rehabilitation adherence, TCM co-intervention intensity, sex, age, and participating hospital site. Performance appeared relatively stable across age, sex, and hospital-site strata, whereas more noticeable variation was observed across baseline functional-capacity, inflammatory-status, rehabilitation-adherence, and TCM co-intervention intensity strata.

In particular, lower performance was observed among patients with markedly reduced baseline 6MWD and among those with lower rehabilitation adherence, suggesting that these subgroups may represent more heterogeneous or less predictable rehabilitation-response patterns. Across diagnosis-based strata, discrimination was highest in the post-viral or long-COVID subgroup and somewhat lower in the COPD subgroup. In the higher inflammatory subgroup, both calibration and discrimination were modestly attenuated relative to the lower inflammatory subgroup, consistent with the stronger negative influence of inflammatory burden observed in the SHAP analyses.

The probability-distribution plots further showed that separation between Responders and Non-responders remained visible across most clinically defined strata, although overlap became more pronounced in subgroups characterized by lower rehabilitation adherence or more severe baseline functional limitation. These subgroup findings should therefore be interpreted as exploratory boundaries of model applicability rather than as confirmatory evidence of effect modification.

Points indicate subgroup-specific ROC-AUC estimates, and horizontal lines indicate 95% confidence intervals. The model maintained acceptable discrimination across major clinical strata, although modest variation was observed across diagnosis, baseline functional capacity, inflammatory status, rehabilitation adherence, and TCM co-intervention intensity. Performance was relatively stable across sex and hospital-site strata. This figure provides a visual summary of the subgroup results reported numerically in Table 7.

Predicted probabilities generated by the final ensemble model are shown for Responders and Non-responders across diagnosis groups, baseline symptom-burden strata, rehabilitation-adherence strata, and TCM co-intervention intensity strata. Clearer separation between Responders and Non-responders indicates better subgroup-level discrimination. The figure shows that probability distributions remained distinguishable across most clinical strata, although overlap was more pronounced in subgroups characterized by lower rehabilitation adherence or more severe baseline functional limitation.

## 4 Discussion

This study developed and internally validated an interpretable stacking ensemble model to evaluate rehabilitation outcomes in patients with respiratory diseases receiving TCM-integrated interventions, using a multicenter real-world retrospective cohort from four tertiary hospitals (12, 13). From a methodological perspective, the study followed core principles for clinical prediction-model development, including prespecified outcome definitions, harmonized assessment time points, strict leakage-prevention procedures, training-set-only preprocessing and feature selection, internal cross-validation, and independent hold-out testing (14). These design features strengthen the credibility of the predictive estimates and reduce the likelihood that the reported performance was driven primarily by overfitting or information leakage. At the same time, the present work addressed a clinically complex setting in which rehabilitation response is shaped by multiple interacting biological, behavioral, and treatment-related factors. In this context, a flexible ensemble framework is better suited than a purely linear approach to capture heterogeneous response patterns across patients (15). Consistent with this rationale, the final stacked model achieved stronger overall performance than the individual candidate models and provided the most balanced profile across discrimination and calibration in the independent test set (16).

The model results suggest that rehabilitation response was jointly associated with baseline functional status, symptom burden, inflammatory activity, and intervention intensity. Among the most influential predictors, baseline 6-min walk distance (6MWD), baseline COPD Assessment Test (CAT) score, and neutrophil-to-lymphocyte ratio (NLR) captured three clinically meaningful dimensions of rehabilitation readiness: exercise capacity, symptom severity, and systemic inflammatory burden (17). These findings are consistent with the broader rehabilitation literature, in which patients with better preserved baseline function and lower symptom or inflammatory burden generally show greater potential for short-term improvement. Importantly, TCM-related variables, including TCM exercise frequency, Herbal Days, and related intervention-intensity measures, were incorporated into the same predictive space rather than being treated as loosely described background treatments. This allowed their contributions to be quantified alongside conventional rehabilitation indicators and provided a more structured basis for evaluating integrated TCM-Western rehabilitation pathways. In practical terms, the findings support the clinical plausibility that TCM intervention intensity may contribute meaningfully to rehabilitation response when interpreted together with baseline disease status and adherence-related features (18)˒ (19).

A further strength of the study lies in the use of SHapley Additive exPlanations (SHAP) to translate model output into clinically interpretable attribution patterns. The combination of global importance ranking, dependence analysis, and individual-level explanation made it possible to move beyond a black-box prediction framework and examine how the model reached its estimates in specific clinical contexts. The nonlinear patterns observed in the dependence plots were particularly informative. For example, the marginal gains associated with better baseline function or higher TCM exercise frequency did not appear to increase indefinitely, but rather showed attenuation at higher levels. This pattern is clinically plausible and may reflect the existence of an “effective window,” beyond which additional intensity yields smaller incremental benefit. Such findings are relevant because they suggest that prediction may support not only static classification of likely response, but also more adaptive rehabilitation management, including follow-up prioritization, intensity adjustment, and allocation of rehabilitation resources according to individualized predicted benefit (20).

The exploratory subgroup analyses also add an important layer of interpretation. Although subgroup findings should not be overread as confirmatory evidence, the final model maintained acceptable discrimination across diagnosis groups, symptom-burden strata, functional-capacity strata, adherence levels, TCM co-intervention intensity levels, and participating hospital sites. This overall consistency suggests that the framework remained reasonably robust under heterogeneous real-world conditions. At the same time, performance attenuation in patients with markedly reduced baseline 6MWD, higher inflammatory burden, or lower rehabilitation adherence indicates that prediction becomes more difficult in clinically unstable or behaviorally variable subgroups. These patterns are also clinically meaningful, because they imply that uncertainty in prediction may itself reflect more heterogeneous rehabilitation trajectories. In this sense, the subgroup results are useful not as proof of universal transportability, but as an early indication of where the model may perform more reliably and where caution is still needed in interpretation.

Several limitations should be acknowledged. First, despite the multicenter design, the present study remains an internally validated retrospective analysis rather than a true external validation study; therefore, the transportability of the model to other regions, institutions, and clinical workflows remains to be established. Second, although rigorous preprocessing and leakage-prevention procedures were implemented, residual bias related to case-mix variation, unmeasured confounding, documentation quality, and missing-data structure cannot be fully excluded in retrospective real-world data. Third, the primary endpoint focused on short-term end-of-course rehabilitation response, which improves temporal consistency for model development but does not capture longer-term maintenance of benefit. Fourth, although SHAP enhances interpretability, explanatory attribution should not be equated with causal inference. Future work should therefore prioritize true external validation, prospective or longitudinal evaluation, and decision-curve analysis to determine whether the model yields clinically meaningful net benefit in real deployment settings. Such steps would strengthen the translational pathway from integrated TCM-Western rehabilitation data to individualized respiratory rehabilitation planning (21).

## 5 Conclusion

This study developed and internally validated an interpretable stacking ensemble framework for predicting short-term rehabilitation outcomes in patients with respiratory diseases receiving TCM-integrated interventions. In the independent test set, the final ensemble model demonstrated stronger overall performance than the individual candidate algorithms and achieved a favorable balance between discrimination and calibration. The findings indicate that rehabilitation response was shaped not only by baseline functional capacity, symptom burden, and inflammatory status, but also by intervention-related variables, including TCM exercise frequency, treatment duration, and adherence-associated features.

By incorporating SHAP-based explainability, the study further translated model predictions into clinically interpretable evidence at both the global and individual levels, thereby improving transparency in the evaluation of integrated rehabilitation strategies. Although external validation is still required, the proposed framework provides a data-driven basis for more individualized rehabilitation assessment and may offer practical value for supporting personalized planning within TCM-Western integrated respiratory rehabilitation pathways (22). Recent evidence on traditional Chinese medicine rehabilitation exercise and research trends in TCM therapies for COPD further supports the relevance of structured TCM-based rehabilitation within integrated respiratory care (23, 24).

## Data Availability

The raw data supporting the conclusions of this article will be made available by the authors, without undue reservation.
